# Key Issues in Designing Long-term Care Systems: A Response to Recent Commentaries

**DOI:** 10.34172/ijhpm.2020.42

**Published:** 2020-03-18

**Authors:** Naoki Ikegami

**Affiliations:** ^1^St. Luke’s International University, Tokyo, Japan.; ^2^Keio University, Tokyo, Japan.

I was delighted to receive nine commentaries to my editorial because they expanded and deepened the understanding of the complex issues involved in designing and implementing the long-term care (LTC) system.^1^ In this response, I have focused on financing and allocating resources because the commentaries could be grouped into these two themes.

## Financing Long-term Care

The aging of the population has been the driving force for expanding LTC. Aging leads to a gradual decline in ADL (activities of daily living such as bathing, dressing, and eating) and IADL (instrumental activities of daily living such as money management, preparing meals, and cleaning rooms) function.^2,3^ The prevalence of Alzheimer’s disease also increases with age.^4^ At the same time, the amount of informal support tends to decline. Loneliness and social isolation could be mitigated by developing social networks and community activities. However, unless LTC services are readily accessible, the major burden would be thrust on the healthcare system that has not been designed to meet LTC needs. Hospitals must discharge the patient after the acute phase has been treated to prevent patients from becoming “bed-blockers.” But if they are discharged without adequate support, they could be readmitted following another crisis. Thus, the government would have to commit itself to providing LTC if only to maintain the efficiency of the healthcare system. This pressure will be felt if the government is committed to providing universal healthcare coverage.

In the United States, Medicare provides universal health coverage for those 65 and over, but LTC is only available from means-tested, state-managed Medicaid. Efforts to expand private insurance have not been successful. As Feng and Glinskaya point out, less than 10% are enrolled in private insurance and is imploded to near collapse.^5^ Private insurance is not suited to finance LTC because the risk increases exponentially with age while the capacity to pay premiums decreases with age. In countries that are contemplating the expansion of publicly financed LTC, there are two options: a tax-based program or a social-insurance-based entitlement program. In the former, the level of services would be more flexibly defined and adjusted to the fiscal space, while it would be more rigidly defined in the latter. However, the method for financing LTC and its scope tends to be path dependent.

Germany and Japan chose social insurance for LTC mainly because this was how they financed healthcare. Benefits were set more generously in Japan because healthcare had been made free for elders in 1973 which led to a de facto delivery of LTC in hospitals and because a decision had been made in 1989 to expand social services in order to win back votes for the ruling party. After the implementation of LTC Insurance, benefits have subsequently been cut to contain costs. On the other hand in Germany, benefits were initially set at a parsimonious level in 1995-1996 but were later made more generous in 2015-2017.^6^ However, the basic structure has remained the same in both countries. This stable status contrasts with the drastic cuts made in Spain’s tax-based program described by Pozo-Rubio and Jimenez-Rubio although the economic crisis must also be taken into consideration.^7^

There are other ways to finance LTC such as the Swiss individual capital-funded occupational retirement fund as described by Eling.^8^ However, this method would not be feasible in countries where pensions are funded on a pay-go basis. Phua et al note that LTC can be financed by saving funds and superannuation.^9^ However, this builds on the method used in healthcare which would make it difficult for most countries to adopt. Finally, I agree with Geyer that private financing has a much greater role in LTC than in healthcare but I reiterate that it would be methodologically very difficult to compile and compare private LTC expenditures across countries.^10^ For example, the LTC cost component of luxury retirement homes and living-in domestic servants would be difficult to estimate.

## Allocating Long-term Care Benefits

The first issue is whether LTC benefits should be in the form of cash or services. Cash benefits provide the maximum freedom. The money could be used to privately hire aides or to compensate the family member providing care. However, for the latter, the hourly amount would be below minimum wages and could chain the family member, usually female, to the caring role. This was why feminist groups in Japan successfully opposed the introduction of cash benefits so that benefits have been de facto limited to the services delivered by certified provider agencies.^11^ This restriction was slightly relaxed in 2015 to include non-certified providers such as volunteer organizations for community services for those in light care levels.

However, benefits cannot be neatly dichotomized into cash and service. Nadash has noted that some states in the United States do not allow the use of the cash benefit to pay a family member.^12^ Thus, there appears to be a continuum in the form of benefits from only in-kind services, to de facto vouchers that are restricted to purchasing services (so as to increase formal LTC workers), to cash with no restrictions on use. Note that offering a choice of cash or services would make the allocation process more regressive because those with low income are more likely to choose cash, which tends to have a lower amount than that for services. Parenthetically, care allowances are usually not considered as LTC expenditures but they decrease the government’s fiscal space to finance LTC services.

Second is the allocation of resources among light to heavy care users. Gori in his study of LTC in seven OECD countries noted that, in community care, there is a general trend to expand coverage (covering more people) than intensity (more resources allocated to each individual), but in community care, it is the reverse.^13^ This would appear to be a logical development because expanding coverage in community care will increase public support for LTC while costs for recipients in institutional care are higher than community care. Parenthetically, public expenditures for institutional care should include the cost of bed and board which may not be covered by LTC Insurance but by public assistance.

Third is whether the expansion should be focused on community care or institutional care. There seems to be a widely shared belief that aging in place in the community is always better than in an institution.^14^ However, the lower costs for community care may have been achieved at the cost of a lower quality of life for the family care provider. The cost of providing formal services in community settings would be higher because care workers require time to visit individual homes. However, this division of LTC into community care and institutional care has become obsolete because of the development of retirement communities and assisted living. In Japan, designated “housing with services for elders” may have a day care center, a visiting care worker office and a visiting nurse station on the ground floor. The number of elders in this and other types of quasi-institutional settings such as group homes has increased so that they equal the number in the traditional institution settings.^15^

Fourth is whether benefits should be restricted to elders or be made available to all ages. This may be more about principles because even if LTC were made available to all, most would be allocated to those 80 over as Okma and Gusmano note.^16^ However, there may be more public support if the program were targeted on elders. In Japan, there was a consensus on the need to meet the challenges of the rapidly aging society which was instrumental in implementing the LTC Insurance. In the program design, if the LTC program were to cover all ages, opportunities for job training must be made available so as to meet the needs of those with physical and mental disability.

Finally is whether LTC programs should be introduced early or late. I had advocated introducing early before ad hoc decisions are made that would lead to disparity among the programs and across local areas. However, Alders and Schut note that although the Netherlands introduced LTC Insurance at an early stage, its current level of expenditures is high.^17^ This may be because the benefit level set by the AWBZ (Algemene Wet Bijozondere Ziektekosten, general law on exceptional medical expenses) covered the full costs of nursing homes. This generous level was later expanded to include other services. To mitigate such continuous benefit expansion, those currently receiving benefits could be grandfathered-in, but new standards should apply to those who are evaluated after the reform.

It was not possible to analyze in depth the complex issues involved in designing LTC systems in this brief response. Ideally, the quality of life of the recipient and of the family care provider should be evaluated together with the costs. But comparing just the cost of LTC in the publicly financed sector was a major task in itself.^18^ What I have attempted to do is to present my views on why LTC should be publicly financed and benefits clearly defined. Decisions to opt for cash or service benefits, to prioritize heavy care or include light care, and to introduce the program early or late must be left to the policy-makers of each country. What is certain is that the need for LTC will continue to increase with the aging of the society.

## Ethical issues

Not applicable.

## Competing interests

Author declares that he has no competing interests.

## Author’s contribution

NI is the single author of the paper.
